# Large Eddy Simulation of a Bluff Body Stabilised Premixed Flame Using Flamelets

**DOI:** 10.1007/s10494-018-9948-9

**Published:** 2018-08-15

**Authors:** James C. Massey, Ivan Langella, Nedunchezhian Swaminathan

**Affiliations:** Department of Engineering, University of Cambridge, Trumpington Street, Cambridge, CB2 1PZ UK

**Keywords:** Turbulent premixed flames, Large Eddy Simulation (LES), Flamelets, Bluff body, Flame stabilisation

## Abstract

Large Eddy Simulations of an unconfined turbulent lean premixed flame, which is stabilised behind a bluff body, are conducted using unstrained flamelets as the sub-grid scale combustion closure. The statistics from the simulations are compared with the corresponding data obtained from the experiment and it is demonstrated that the experimental observations are well captured. The relative positioning of the shear layers and the flame brush are analysed to understand the radial variations of the turbulent kinetic energy at various streamwise locations. These results are also compared to confined bluff body stabilised flames, to shed light on the relative role of incoming and shear driven turbulence on the behaviour of the flame brush and the turbulent kinetic energy variation across it.

## Introduction

Bluff body burners are often used in practical combustion systems, such as stationary gas turbines, industrial burners and afterburners. The hot recirculation zone behind the bluff body offers a simple mechanism for flame stabilisation by providing a continuous supply of heat to ignite the incoming fuel and air mixture. However, the size and shape of the recirculation zone influences the performance of these combustion systems and these attributes depend on the incoming flow rate, equivalence ratio and bluff body geometry. Such configurations make it feasible to investigate flame blow-off conditions as a function of incoming flow rates and equivalence ratio for a given bluff body geometry through well controlled experiments, as done in [1]. This fundamental information is required at the design stage of combustors with the purpose of operating under lean-burn conditions, since efficiency and environmental benefits can be achieved. It is well known that lean flames are susceptible to extinction and combustion instabilities that may lead to the occurrence of flame blow-off, which is typically treated as the complete extinction of the flame [2–4]. The physical processes and their interactions governing these phenomena are highly unsteady, where it has been mainly hypothesised in previous studies that the competing effects of convection and chemical reaction (combustion) lead to flame blow-off. These competing effects may be governed by a number of physical processes, such as large scale entrainment of reactants into the recirculation zone amongst other phenomena. It has also been noted in [5] that the process of flame blow-off and a general physical mechanism for its occurrence are not fully understood.

Large Eddy Simulation (LES) has emerged as a powerful and insightful computational approach, due to its potential to capture unsteady phenomena, such as ignition and blow-off. With this approach, the dynamic scales of turbulence and scalar fields are resolved up to a cut-off scale, Δ, and the remaining sub-grid scale (SGS) processes are modelled. Turbulent combustion is typically an SGS phenomenon and therefore requires modelling. Various models have been proposed in past studies and are reviewed in [6–10]. Out of these models, the flamelet approach is the simplest one and it has been demonstrated recently that this approach can be used for capturing multi-regime turbulent premixed combustion; this was previously achieved by considering a bluff body stabilised turbulent premixed flame within a confined chamber [11].

A long term objective is to predict flame blow-off using a flamelet approach. However, the specific focus of this work is to demonstrate, as a first step, that an unstrained flamelet model can accurately capture the stabilisation of flames far from blow-off conditions, along with the various flame and flow attributes of the bluff body stabilised open flame. This is achieved by simulating the bluff body stabilised methane-air flames investigated experimentally in [1], which differ substantially from the flames considered in the study by Langella et al. [11]. The experimental flames investigated in [11] were confined within a rectangular duct that contained a turbulence generator upstream of the bluff body base. Thus, the turbulence experienced by those flames came from both incoming and shear generated turbulence [12–16], whereas the flames investigated by Kariuki et al. [1] experienced mostly shear driven turbulence, since a turbulence generator was absent in the burner configuration. An additional complexity also arose from the entrainment of surrounding air because the flames were open to atmospheric conditions. The entrainment effects are expected to be small for flames far from blow-off but could play important roles for flames close to blow-off because of the potential dilution of an already weaker mixture for the flames closer to blow-off, as the equivalence ratios are lower. However, the flame features could be quite different between open and confined flames, specifically near the bluff body because there is no incoming turbulence in the open flames considered for this study.

In light of the results presented by Langella et al. [11], the aim of this study is to test the applicability of the unstrained premixed flamelet closure as SGS combustion closure for modelling the open bluff body stabilised turbulent premixed flame that is furthest from blow-off. In addition, this study highlights the differences in the spatial evolution of the shear layers and the flame brush for the open flame studied here and for confined bluff body stabilised flames. This paper is organised as follows. The experimental test case is described in the next section, followed by its LES set-up in Section 3. The results of the simulations are shown in Section 4, along with some interesting features of the open flame. The key findings and conclusions are summarised in the last section.

## Test Case and Flame Conditions

**Fig. 1:**
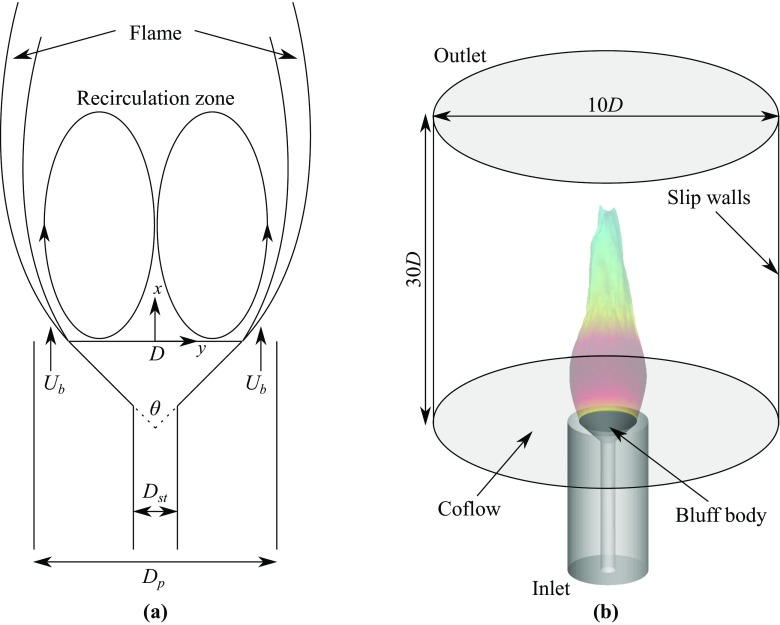
Schematic of the open bluff body burner investigated in [1] **a** and its computational model **b**

The open bluff body burner used for this study was investigated experimentally in [1] and this burner is based on that developed in [17]. The schematic of this burner is shown in Fig. 1a and the computational model, shown in Fig. 1b, will be discussed in Section 3.3. The burner had a conical bluff body with a cone angle of 90^∘^. This body was mounted on a circular rod of diameter *D*_*st*_ = 6.35 mm and the bluff body was fitted within a concentric pipe of diameter *D*_*p*_ = 35 mm. The bluff body base had a diameter of *D* = 25 mm and was exposed to atmospheric air, where there was no external flow present. The relevant parameters of the burner are listed in Table 1. A premixed methane-air mixture at ambient conditions with an equivalence ratio of *ϕ* = 0.75 entered through the annular gap, shown in Fig. 1a. This gave a bulk mean velocity of *U*_*b*_ = 21.6 m/s at the base of the bluff body, which is used as the reference velocity for this study. There was not a turbulence generating device present upstream of the bluff body base and therefore, only the turbulence produced through shear is present, which originates from the trailing edge of the bluff body.

**Table 1:** Physical parameters of the bluff body burner used in [1]

Parameter	Value	Description
*D*	25 mm	Bluff body diameter
*T*_*u*_	298 K	Inlet temperature for methane-air mixture
*U*_air_	0.1 m/s	Coflow velocity (see Fig. 1b)
*U*_*b*_	21.6 m/s	Reference bulk velocity at the bluff body
		base
*ϕ*	0.75	Methane-air equivalence ratio

Measurements of four flames that approached blow-off conditions were taken in the experiments by Kariuki et al. [1]. Blow-off conditions were approached by gradually decreasing the equivalence ratio from *ϕ* = 0.75 (A1) to a value just prior to blow-off (A4), whilst maintaining the same value for *U*_*b*_. The measurements were made using Particle Image Velocimetry (PIV) for velocities, and OH^∗^ chemiluminescence and OH-PLIF techniques to identify the flame location and shape, as described in [1]. This flame has been simulated in past studies using conditional moment closure [18], the Eulerian stochastic field method [19] and a finite rate chemistry approach [20]. As mentioned in Section 1, the flame furthest away from the blow-off (A1) is of interest for this work and is simulated using LES and the combustion closure that is described in the next section.

## Large Eddy Simulation

### Governing equations

The conservation equations for mass, momentum and total enthalpy (energy) are solved along with two additional equations for combustion. The Favre-filtered form of these equations are used because of strong density variations in the flow arising from the heat release from combustion. The additional equations are for the Favre-filtered reaction progress variable and its SGS variance. These five equations are written as

Mass:

1 $$ \frac{\partial \overline{\rho}}{\partial t} + \frac { \partial \overline{ \rho } { \widetilde{ U } }_{j } }{ \partial { x }_{j } } = 0 $$

Momentum:

2 $$ \overline{ \rho } \frac{ \mathrm{ D } { \widetilde{ U } }_{i } }{ \mathrm{ D } t } = - \frac { \partial \overline{ p } }{ \partial { x }_{i } } + \frac { \partial { \overline{ \tau }}_{i j } }{ \partial { x }_{j } } - \frac{ \partial }{ \partial { x }_{j } } \left( \overline{ \rho { U }_{j } { U }_{i } } - \overline{ \rho } { \widetilde{ U } }_{j }{ \widetilde{ U } }_{i } \right) $$

Total enthalpy:

3 $$ \overline{ \rho } \frac{ \mathrm{ D } \widetilde{ h } }{ \mathrm{ D } t } = \frac { \partial }{ \partial { x }_{j } } \left( \overline{ \rho \alpha } \frac { \partial \widetilde{ h } }{ \partial { x }_{j } } \right) - \frac{ \partial }{ \partial { x }_{j } } \left( \overline{ \rho { U }_{j } h } - \overline{ \rho }{ \widetilde{ U } }_{j } \widetilde{ h } \right) $$

Reaction progress variable:

4 $$ \overline{ \rho } \frac{ \mathrm{ D } \widetilde{ c } }{ \mathrm{ D } t } = \overline{ \dot { \omega } } + \frac { \partial }{ \partial { x }_{j } } \left( \overline{ \rho \mathcal{ D } } \frac { \partial \widetilde{ c } }{ \partial { x }_{j } } \right) - \frac{ \partial }{ \partial { x }_{j } } \left( \overline{ \rho { U }_{j } c } - \overline{ \rho }{ \widetilde{ U } }_{j } \widetilde{ c } \right) $$

SGS variance:

5 $$\begin{array}{@{}rcl@{}} \overline{ \rho } \frac{ \mathrm{ D } { \sigma }_{c , \mathrm{ sgs } }^{2 } }{ \mathrm{ D } t } & \simeq& \frac { \partial }{ \partial { x }_{j } } \left( \left[ \overline{\rho \mathcal{ D } } + \overline{ \rho } \frac{ { \nu }_{T } }{ { \mathrm{ Sc } }_{T } } \right] \frac { \partial { \sigma }_{c , \mathrm{ sgs } }^{2 } }{ \partial { x }_{j } } \right) \\ && + 2 \left( \overline{ \dot{ \omega } c } - \overline{ \dot { \omega } } \widetilde{ c } \right) - 2 \overline{ \rho } { \widetilde{ \chi } }_{c , \mathrm{ sgs } } + 2 \overline{ \rho } \frac{ { \nu }_{T } }{ { \mathrm{ Sc } }_{T } } \left( \frac { \partial \widetilde{ c } }{ \partial { x }_{j } } \frac { \partial \widetilde{ c } }{ \partial { x }_{j } } \right) \end{array} $$

The density and velocity are denoted using *ρ* and *U* respectively, and the total enthalpy is the sum of the sensible and chemical enthalpies, which is denoted as *h*. The notation D/D*t* = *∂*/*∂t* + *U*_*j*_*∂*/*∂x*_*j*_ denotes the substantial derivative. The progress variable, *c*, can be defined using either temperature or species mass fractions. For this study, the CO and CO_2_ mass fractions are used, and hence the progress variable is defined as $c = ({ Y }_{\mathrm { CO } } + { Y }_{{ \mathrm { CO } }_{2 } } ) / { ({ Y }_{\mathrm { CO } } + { Y }_{{ \mathrm { CO } }_{2 } } ) }_{b }$, where the subscript *b* denotes the burnt mixture value. This takes a value of zero and unity in the unburnt and burnt mixtures respectively. The symbols *α* and $\mathcal { D }$ respectively denote the molecular thermal diffusivity of the mixture and the mass diffusivity of *c*. The SGS stresses, denoted by the last term of Eq. 2, are modelled using the eddy-viscosity concept [9], where the dynamic Smagorinsky approach [21, 22] is used to model the sub-grid eddy viscosity, *ν*_*T*_. The sub-grid scalar fluxes in Eqs. 3 to 5 are modelled using gradient hypotheses, where the turbulent Schmidt number, Sc_*T*_, is calculated dynamically [22]. The filtered reaction rate, $\overline { \dot { \omega } }$, in Eq. 4 and the reaction-related source terms in Eq. 5 for the SGS variance, ${ \sigma }_{c , \mathrm { sgs } }^{2 }$, achieve closure by the combustion modelling described in Section 3.2. The sub-grid scalar dissipation rate, ${ \widetilde { \chi } }_{c , \mathrm { sgs } }$, is influenced by both combustion and turbulence in premixed flames and thus, its modelling should include contributions from these two processes. One such model was developed [23] and tested [24–26] in past studies and this model is briefly discussed next, along with the combustion modelling used for this study. The importance of transporting the SGS variance and the closure models used within its transport equation have been discussed in previous studies [11, 24] and the behaviour of the SGS variance in bluff body flames is discussed in [11].

### Combustion closure

The combustion modelling used here is based on the unstrained premixed flamelet concept. The flamelet concept assumes that the flame is thin, so that turbulence cannot affect its inner structure. This allows the thermochemistry to be decoupled from turbulence and the flame can be seen as a series of one-dimensional thin structures, known as flamelets. The profile of the reaction progress variable for each flamelet is identical to the profile of a laminar flame. The wrinkling of the flame is accounted for by a presumed PDF, where the progress variable and its SGS variance are used to track the reaction. Numerous previous studies have tested this approach, which include laboratory scale flames with Reynolds-Averaged Navier-Stokes (RANS) [27–30] and unsteady RANS (URANS) methodologies for laboratory scale flames [31] and for a practical burner [32, 33]; this model for LES has been developed and tested in [11, 24, 34]. The model is discussed briefly here and a more detailed description can be found in the references cited above.

The filtered reaction rate is given by

6 $$ \overline{ \dot{ \omega } } = {\int}_{0 }^{1 } \dot{ \omega } \left( \zeta \right) P \left( \zeta ; \widetilde{ c } , { \sigma }_{c, \mathrm{ sgs } }^{2 } \right) \mathrm{ d } \zeta = \overline{ \rho } {\int}_{0 }^{1 } \frac{ \dot{ \omega } \left( \zeta \right) }{ \rho } \widetilde{ P } \left( \zeta ; \widetilde{ c } , { \sigma }_{c , \mathrm{ sgs } }^{2 } \right) \mathrm{ d } \zeta , $$

where $\widetilde { P } (\zeta ; \widetilde { c } , { \sigma }_{c,\text {sgs}}^{2 } )$ is the density-weighted sub-grid PDF of the reaction progress variable, and its sample space variable is denoted *ζ*. The flamelet reaction rate and mixture density are denoted using $\dot {\omega } \left (\zeta \right )$ and *ρ* respectively. The shape of the sub-grid PDF is assigned using a beta function for given values of *c* and ${ \sigma }_{c, \mathrm { sgs } }^{2 }$, which are obtained from their respective transport equations, Eqs. 4 and 5.

The modelling of the reaction related term in the SGS variance equation follows a similar procedure and is written as

7 $$ \overline{ \dot { \omega } c } = \overline{ \rho } {\int}_{0 }^{1 } \left( \frac{ \dot{ \omega } \zeta }{ \rho } \right) \widetilde{ P } \left( \zeta \right) \mathrm{ d } \zeta $$

The above two sources (integrals) are precomputed using an unstrained premixed laminar flame calculation and are tabulated as a function of $\widetilde { c }$ and ${ \sigma }_{c, \mathrm { sgs } }^{2 }$, which is used in the LES. The sub-grid dissipation rate, which is the fourth term in Eq. 5, is modelled using an algebraic closure [23], which has been tested thoroughly in past studies [11, 24, 35]. This expression is

8 $$ { \widetilde { \chi } }_{c, \mathrm{ sgs } } = \mathcal{ F } \left[ 2 { K }_{c } \frac{ { s }_{L } }{ { \delta }_{\mathrm{ th } } } + \left( { C }_{3 } - \tau { C }_{4 } { \mathrm{ Da } }_{{\Delta} } \right) \left( \frac{ 2 { u }_{{\Delta} }^{\prime } }{ 3 {\Delta} } \right) \right] \frac{ { \sigma }_{c , \mathrm{ sgs } }^{2 } }{ { \beta }_{c } } $$

where the function $\mathcal { F } = 1 - \exp (- 0.75 { {\Delta } }^{+ } )$ ensures that the SGS dissipation rate goes to zero when the filter width approaches zero. The normalised filter width is Δ^+^ = Δ/*δ*_th_, where the laminar flame thickness is *δ*_th_ = 0.55 mm, and the laminar flame speed is *s*_*L*_ = 0.26 m/s for the methane-air mixture used in the test case described earlier. The thermochemical parameter is *K*_*c*_ = 0.79*τ*, where *τ* is the temperature rise across the flamelet, which is normalised by the unburnt mixture temperature, *T*_*u*_. The other parameters are defined as ${ C }_{3 } = 1.5 \sqrt { { \mathrm { Ka } }_{{\Delta } } } / (1 + \sqrt { { \mathrm { Ka } }_{{\Delta } } } )$, *C*_4_ = 1.1/(1 + Ka_Δ_)^0.4^, where ${ \mathrm { Ka } }_{{\Delta } }= { ({ u }_{{\Delta } }^{\prime } / { s }_{L } ) }^{3/2 } { ({ \delta }_{\mathrm { th } } / {\Delta } ) }^{1/2 }$, and ${ \mathrm { Da } }_{{\Delta } } = { s }_{L } {\Delta } / ({ u }_{{\Delta } }^{\prime } { \delta }_{\mathrm { th } } )$. The symbol ${ u }_{{\Delta } }^{\prime }$ is a SGS velocity scale requiring a closure and is modelled using ${ u }_{{\Delta } }^{\prime } = { C }_{q } { \sum }_{j } \vert \widetilde { U }_{j } - \widehat { \widetilde { U } }_{j } \vert $ [36], where $\widehat { \widetilde { U } }_{j }$ is the velocity field using a Gaussian test filter during the LES. The test filter width is $\widehat { {\Delta } } \simeq 2 {\Delta }$, where the LES filter width is estimated as ${\Delta } = { \mathcal { V } }^{1/3 }$, with $\mathcal { V }$ being the volume of the computational cell.

It has been established in past studies that the above parameters and their values for Eq. 8 are closely connected to certain physical aspects of the scalar dissipation rate transport and elaborate detail can be found in [23, 37, 38]. The term ${ \sigma }_{c ,\mathrm { sgs } }^{2 } / { \beta }_{c }$ is related to the influence of flame curvature, which is induced by wrinkling. The parameter *β*_*c*_ is therefore scale dependent, which is evaluated using a dynamic approach described in [25, 35]. Thus, there is no adjustable parameter in this modelling approach, but for the sake of comparison, a static value of *β*_*c*_ = 0.4 is also used for this study.

The Favre-filtered temperature is obtained from the computed total enthalpy according to $\widetilde { T } = { T }_{0 } + (\widetilde { h } - { \widetilde { {\Delta } h } }_{f }^{0 } ) / { \widetilde { c } }_{p }$, where ${ \widetilde { {\Delta } h } }_{f }^{0 }$ and ${ \widetilde { c } }_{p }$ represent the formation enthalpy and specific heat capacity respectively at constant pressure of the gas mixture, and the reference temperature is *T*_0_ = 298.15 K. The mixture density is computed as $\overline { \rho } = \overline { p } \widetilde { M } / { \Re }^{0 } \widetilde { T }$, where $\widetilde { M }$ represents the Favre-filtered mixture molecular mass and *R*^0^ is the universal gas constant. The three thermochemical quantities for the mixture, ${ \widetilde { {\Delta } h } }_{f }^{0 }$, ${ \widetilde { c } }_{p }$ and $\widetilde { M }$, are calculated in a manner similar to Eq. 6, described in detail in [39]. These quantities, along with the reaction-related source terms, are tabulated as a function of $\widetilde { c }$ and ${ \sigma }_{c, \mathrm { sgs } }^{2 }$. This look-up table has 101 and 51 evenly distributed points for $\widetilde { c }$ and *σc*,sgs2 respectively for this study. The flamelet is computed using the PREMIX code [40] and the GRI-Mech 3.0 chemical kinetic mechanism for methane-air combustion.

### Boundary conditions, computational grid and numerical solver

The computational model of the open bluff body burner, discussed in Section 2, is shown in Fig. 1b. As seen in this figure, the computational grid starts at roughly 70 mm upstream of the bluff body base, where a flat velocity profile is prescribed at the inlet to give the required mass flow rate through the burner. This mass flow rate gives the reference bulk mean velocity, *U*_*b*_, at the base of the bluff body, as marked in Fig. 1a. A cylindrical region is added downstream from the bluff body base, as illustrated in Fig. 1b, to represent the boundary entraining the atmospheric air. A small velocity of *U*_air_ = 0.1 m/s is specified at the boundary, marked as “Coflow” in Fig. 1b, to mimic the ambient air entrainment around the bluff body. Adiabatic no-slip wall conditions are imposed on the pipe walls and bluff body, following the previous numerical study [11]. The cylindrical boundary is specified to be a slip wall and for the outlet, the streamwise gradient of all the variables is set to zero.

The variables $\widetilde { c }$ and ${\sigma }_{c, \text { sgs}}^{2}$ are set to be zero for both the inlet and coflow boundaries and the enthalpies for these boundaries are set to be consistent with their temperature and composition. The effect of the entrainment is captured by transporting a passive fluid marker, $\widetilde { Z }$, which is set to be unity in the methane-air stream and zero for ambient air. Following earlier studies [24, 34], the thermochemical property, $\widetilde { \varphi }$, of the mixture is determined using a mixing rule ${ \widetilde { \varphi } }_{\mathrm { mix } } = \widetilde { Z } { \widetilde { \varphi } }_{\mathrm { reac } } + (1 - \widetilde { Z } ) { \varphi }_{ \mathrm { air } }$, where the subscripts “reac” and “air” denote the values of $\widetilde { \varphi }$ taken from the look-up table and air respectively.

A block structured computational grid is used to discretise the computational volume shown in Fig. 1b. The grid consists of approximately 3.6 million hexahedral cells in total, with refinement near the bluff body and in the regions where the shear layers and filtered flames are expected to be present. The minimum cell size in these regions is around 0.2 mm. The wall boundary layers are resolved by placing two cells within the viscous sub-layer, as recommended in a previous study [11].

The code, PRECISE-MB [41], used for this work solves the reacting flow equations along with the combustion modelling, described in Sections 3.1 and 3.2 respectively, using the finite volume methodology. The spatial gradients are calculated using second-order accurate central difference schemes [42]. The discretised equations are time-advanced using a second-order scheme. The Courant-Friedrichs-Lewy (CFL) number is kept below 0.3 by specifying a constant time step of 5 *μ* s. The velocity and pressure coupling is maintained using the SIMPLEC algorithm [43].

All the simulations reported in the next section are run using the Cambridge High Performance Computing Cluster, Darwin. Each node of this cluster has two 2.6GHz eight-core Sandy Bridge E5-2670 processors. The simulations were run using 96cores, which required 36hrs of wall clock time for a simulation over a period of sixteen flow through times. The flow through time is defined as *ℓ*/*U*_*b*_, where *ℓ* is the reference length and is taken as *ℓ* = 150mm. The time-averaged statistics are obtained using the samples collected over the latter half of the simulation time, which are discussed in the next section.

## Results

### Isothermal flow

**Fig. 2:**
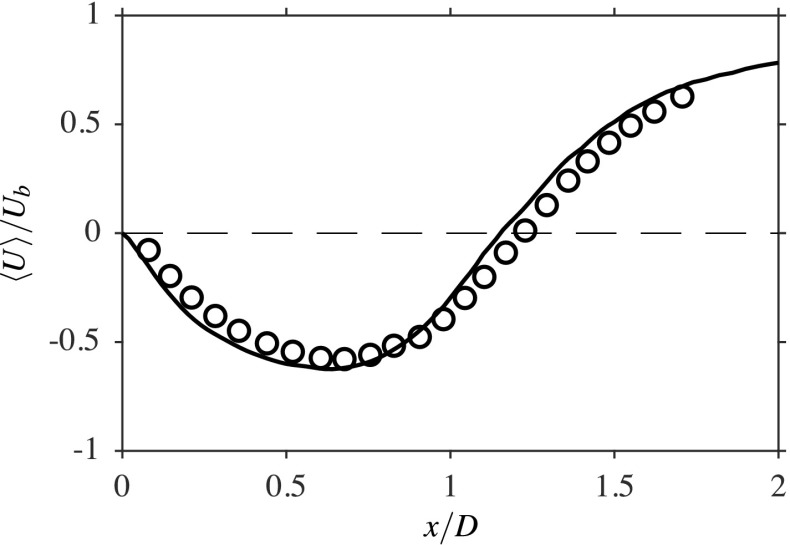
Comparison of the centreline axial velocity from the bluff body base for the isothermal case between the LES (−−−) and the experiment [1] (∘)

Figure 2 compares the computed and measured axial variation of the streamwise velocity along the centreline. The time-averaged value is normalised using the reference velocity, *U*_*b*_, and the axial distance is normalised using the bluff body diameter, *D*. The time-averaged velocity is also averaged azimuthally because of the axisymmetric nature of the averaged flow features and the averaged quantities are denoted using angle brackets in the following discussion; this approach is also used for the flame results, which are discussed in the next subsection. The velocity measurements were obtained using PIV [1]. As shown in this figure, the comparison between the measured and computed values is good. The negative values imply the reverse flow within the recirculation zone and thus, the length of the recirculation zone is given by the *x* distance of the zero crossing of the normalised velocity. The computed value is 1.15*D*, which agrees well with the measured value of roughly 1.22*D*.

**Fig. 3:**
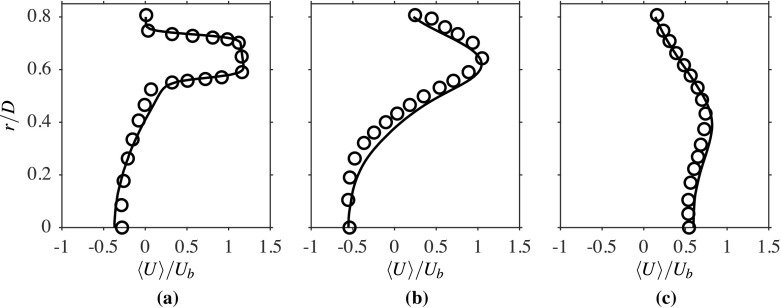
Comparison of computed (−−−) and measured [1] (∘) normalised averaged axial velocity using *U*_*b*_ at **a** *x*/*D* = 0.2, **b** 0.8 and **c** 1.6 for the isothermal case

**Fig. 4:**
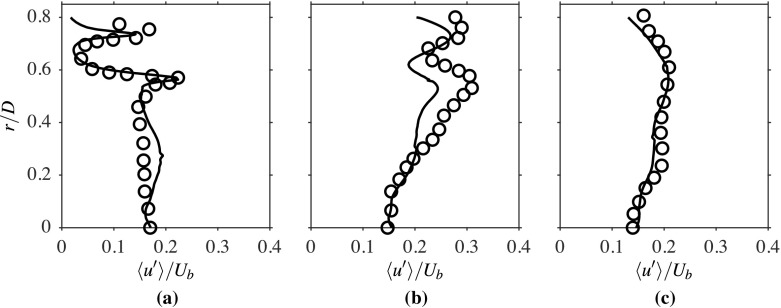
Comparison of computed (−−−) and measured [1] (∘) $\langle { u }^{\prime } \rangle /{ U }_{b }$ at **a** *x*/*D* = 0.2, **b** 0.8 and **c** 1.6, for the isothermal case

The computed radial variations of the time-averaged axial velocity, normalised using *U*_*b*_, is compared to the measurements in Fig. 3 for three streamwise locations. The comparison seen here is good and suggests that the salient features, such as shear layers and peak velocity values of the flow, are well caputured in the computations. The broad peak seen for the location *x*/*D* = 0.2 corresponds to the annular jet region, implying there are two shear layers, namely the inner and outer shear layers. The width of the recirculation zone changes from roughly 0.5*D* at *x*/*D* = 0.2 to around 0.4*D* at *x*/*D* = 0.8. The change in the radial variation with the streamwise distance shows that the width of the shear layers are increasing with *x*. These variations are captured well in the computations, as seen in Fig. 3. Specifically, the recirculation zone size is directly influenced by the turbulence level near the bluff body base because this zone is established by the momentum diffusion caused by the turbulent diffusivity. It is essential to accurately capture the averaged velocity and turbulence statistics variations near the bluff body base, as they control the recirculation zone attributes that help the flame’s stabilisation. As noted in Section 2, the turbulence is shear driven and it is expected that the root mean square (rms) value of the axial velocity fluctuations, *u*^′^, will be larger in the regions with large $\partial \langle \widetilde { U } \rangle / \partial r$, which can be clearly seen in Figs. 3 and 4. This figure compares the computed radial variations of *u*^′^, which is normalised using *U*_*b*_, with measurements for the three axial locations shown in Fig. 3. Both the positions and magnitudes of the two local peaks of *u*^′^/*U*_*b*_ at the location *x*/*D* = 0.2 are captured well in the computations. There is an overall increase in the axial rms velocity further downstream, as seen in Fig. 4b, because of the increase in shear driven turbulence, but there is some small under prediction in the computation for this location. The local peaks are less defined as the shear layers become thicker with axial distance because of turbulent diffusion. The measured values for the location *x*/*D* = 1.6 are well captured in the computation, as seen in Fig. 4c, and this location is outside the recirculation zone (beyond the downstream stagnation point). The comparisions shown between the computed and measured statistics for isothermal flow suggest that the numerical grid used and the computational model is good and this set-up is used for reacting flow, which is discussed next.

### Reacting flow

#### General flame features

**Fig. 5:**
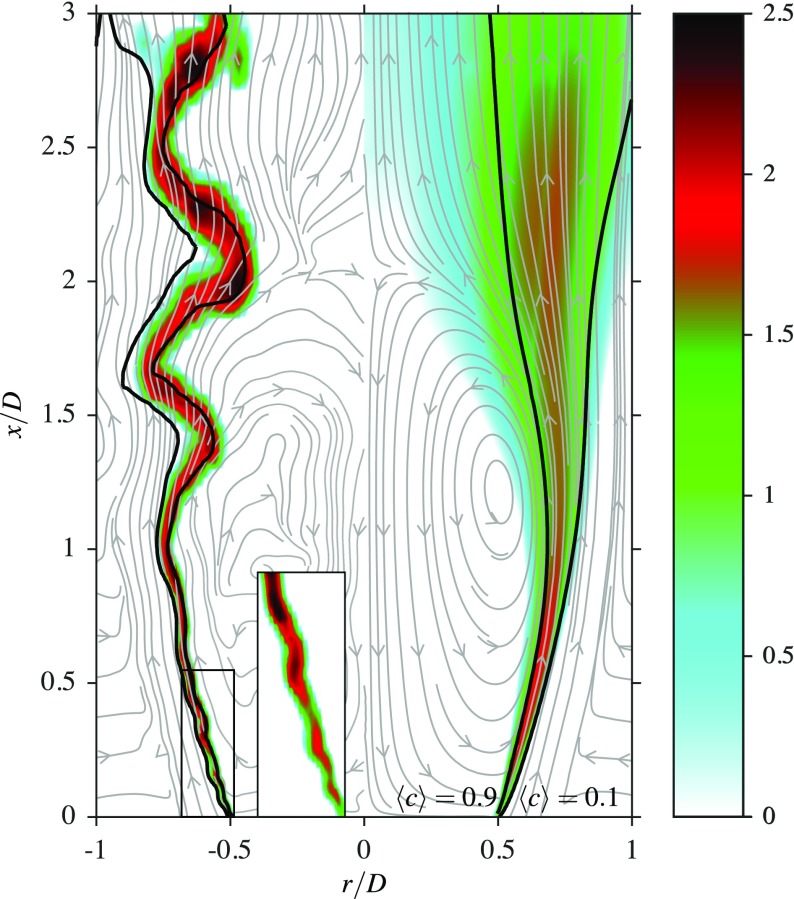
Contours of instantaneous $\log (1000 { \widehat { \mathrm { Da } } }_{{\Delta } } )$ and velocity streamlines are shown in the left half. The right half shows the contours of the time-averaged quantities, $\log (1000 \langle { \widehat { \mathrm { Da } } }_{{\Delta } } \rangle )$ and the corresponding streamlines

**Fig. 6:**
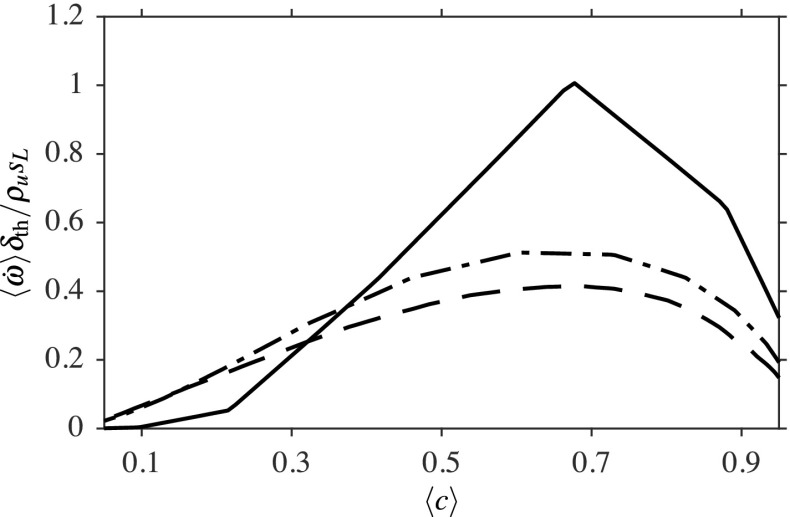
Time-averaged reaction rate across the flame brush at three streamwise locations; *x*/*D* = 0.2 (−−−), 0.8 (− ⋅ −) and 1.6 (−−).

**Fig. 7:**
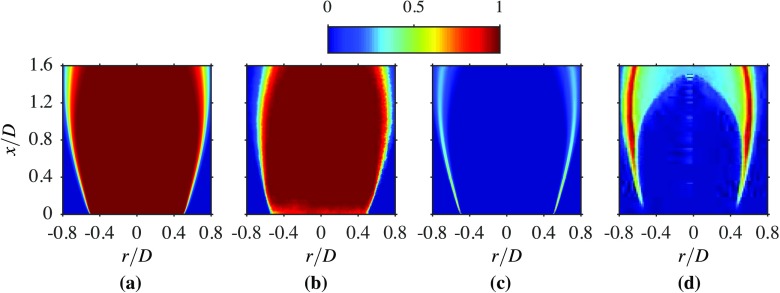
Comparison of the averaged progress variable contours from the LES **a** against the experiment [1] **b**, and averaged reaction rate from the LES **c** against Abel transformed OH^∗^ from [1] **d**

Figures 5, 6 and 7 show qualitative features of the computed flame; the quantitative comparisons will be discussed in the next section. The local SGS Damköhler number, defined as the ratio of the SGS flow time scale, *τ*_sgs_, to the chemical time scale, *τ*_*c*_, can be used to mark the regions with combustion. This dimensionless quantity is given by ${ \widehat { \mathrm { Da } } }_{{\Delta } } = { \tau }_{\mathrm { sgs } } / { \tau }_{c } = \overline { \dot { \omega } } {\Delta } / ({ \rho }_{u } { u }_{{\Delta } }^{\prime } )$. The spatial variation of ${ \widehat { \mathrm { Da } } }_{{\Delta } }$ is shown in Fig. 5, along with the velocity streamlines. The left half of this figure shows the local values of $\log (1000 { \widehat { \mathrm { Da } } }_{{\Delta } } )$ from an arbitrarily chosen snapshot of the data, along with the corresponding streamlines. The right half shows the log of the averaged values, i.e., $\log (1000 \langle { \widehat { \mathrm { Da } } }_{{\Delta } } \rangle )$, along with the streamlines of the averaged flow field. In addition, the isolines of the instantaneous and the averaged progress variable, having values of $\widetilde { c } = 0.1$ and 0.9, are also shown to mark the filtered flame and the flame brush.

The variation of ${ \widehat { \mathrm { Da } } }_{{\Delta } }$ is strong within the instantaneous and averaged reacting regions, as shown in Fig. 5. Larger values are near the bluff body base in the averaged image, while the instantaneous field shows that large values also occur at downstream locations. These results suggest that the combustion is in the corrugated flamelet regime for the region immediately downstream of the bluff body base, as seen in the inset of Fig. 5. The flame becomes thicker for 0.5 ≤ *x*/*D* ≤ 1.5, due to influence of shear layer roll-up because of the Kelvin-Helmholtz instability on the filtered flame. The values of $\langle { \widehat { \mathrm { Da } } }_{{\Delta } } \rangle $, shown on the right of Fig. 5, suggest that the combustion is in the thin reaction zones regime. For *x*/*D* > 2, the combustion is observed to be in the distributed reaction zones regime and therefore it is clear that multi-regime combustion occurs behind a bluff body.

The radial variation of the time-averaged filtered reaction rate is shown in Fig. 6 as a function of the time-averaged progress variable. Thus, this figure shows the variation of the averaged reaction rate across the flame brush. The reaction rates are normalised using *ρ*_*u*_, *s*_*L*_ and *δ*_th_ appropriately and the results are shown for three axial locations. The peak value of this normalised reaction rate is of order unity for the location *x*/*D* = 0.2, suggesting that the combustion is occurring in the corrugated flamelets regime for this location. Moving downstream from this point, the peak value is dropping gradually from unity, suggesting a broadening of the flame. This broadening implies that the combustion regime is changing gradually further downstream from the bluff body base. These results support the observation made in Fig. 5 regarding the combustion regime. The change in the combustion regime is due to the chemistry becoming gradually weaker compared to the shear generated turbulence when moving away from the base of the bluff body.

Figure 7 compares the computed spatial variations of the averaged reaction progress variable and reaction rate with their appropriate measurements. All quantities are normalised to ensure that they vary from zero to unity, as shown in the figure. The progress variable for the experiment is based on the OH measured in the experiment, whereas the progress variable is based on CO and CO_2_ mass fractions for the computations, as noted earlier. The progress variable fields, shown in Fig. 7a and b, suggest that the flame length and width are slightly overestimated in the computations. However, the LES results show a flame shape very similar to that observed in the experiment. This is also supported by the results in Fig. 7c and d, showing the computed averaged reaction rate and the measured OH^∗^ chemiluminescence image respectively. This chemical species is known to be a good surrogate for heat release regions for marking the flame and thus, the reaction rate, which is readily available in the computations, is used for this comparison. Also, the heat release is given by the product of the lower heating value of the fuel and the reaction rate. It can be seen in Fig. 7c that the peak reaction rate is in the vicinity of the bluff body base within the thin layer, as noted while discussing Fig. 5, and this thin layer consists of roughly five to six numerical cells in the radial direction. The peak reaction rate value decreases further downstream because of the broadening of the filtered flame, which is consistent with the SGS Damköhler number variation and filtered reaction rates, shown in Figs. 5 and 6 respectively. These qualitative comparisons suggest that the general features of the bluff body stabilised open flame are captured satisfactorily by the unstrained flamelet combustion closure used for this study. However, quantitative assessments are to be made, which are discussed next.

#### Comparisons with measurements

The experimental investigation in [1] used PIV and planar imaging of OH using laser induced fluorescence techniques and OH^∗^ chemiluminescence. The quantitative comparisons are therefore limited to only velocity statistics, since no temperature or species measurements were obtained on this burner. However, it is said that the flow fields are strongly influenced by combustion and the entrainment effects. Therefore to get good comparisons between the measurements and computational results, the combustion model and its interaction with flow and entrainment effects must be well captured. This is further to the numerical grid requirements, which were validated using the cold flow results in Section 4.1.

**Fig. 8:**
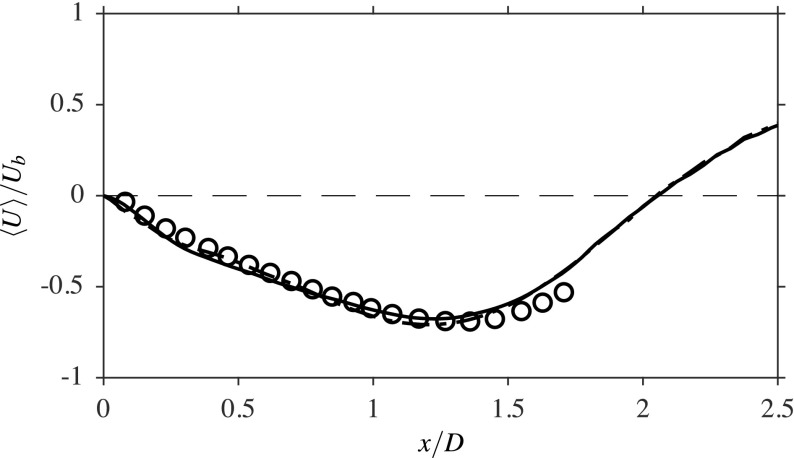
Comparison of the computed (lines) and measured [1] (∘) axial velocities in the flame A1. The results obtained using dynamic (−−−) and static (− ⋅ −) approaches for *β*_*c*_ (see Eq. 8)

**Fig. 9:**
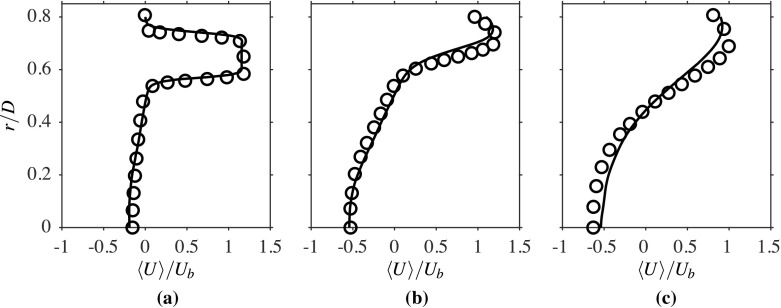
Computed (−−−) and measured [1] (∘) radial variations of averaged axial velocity at **a***x*/*D* = 0.2, **b** 0.8 and **c** 1.6 for the reacting case

Figure 8 compares the measured and computed averaged streamwise velocity variations along the centreline of the burner, where the velocity is again normalised using *U*_*b*_. The measurements do not span the entire recirculation zone length, however the comparison shown with the available data is good. As expected, the presence of combustion influences the recirculation zone length, which is seen to increase from 1.15*D* in the isothermal case to 2.02*D* in the reacting case. This length is close to the value reported in the experiment, which was roughly 2*D* [1]. As discussed in Section 3.2, the model parameter *β*_*c*_ in Eq. 8 can be evaluated dynamically or prescribed with a static value. For the sake of comparison, the axial variation of the normalised averaged streamwise velocity is obtained using the dynamic and static approaches; these are shown in Fig. 8. A static value of *β*_*c*_ = 0.4 was chosen *a posteriori* using the results from a dynamic procedure. It is seen that the results are almost identical, which is due to the careful selection of the value for *β*_*c*_. This choice is not always possible and the use of dynamic procedure is preferred, despite the small additional computational cost that is required. Therefore, all of the results shown and discussed use the dynamic approach. The radial variations of the computed and measured axial velocity are compared in Fig. 9 for the same three streamwise locations that were considered for the isothermal case in Fig. 3. The LES results at all three locations show a good agreement with the measurements [1]. These variations are similar to those observed for non-reacting flow, except the peak magnitude is larger for the reacting case, due to the heat release effects. The comparison seen in Fig. 9 suggests that the locations of both the inner and outer shear layers are well captured in the LES.

**Fig. 10:**
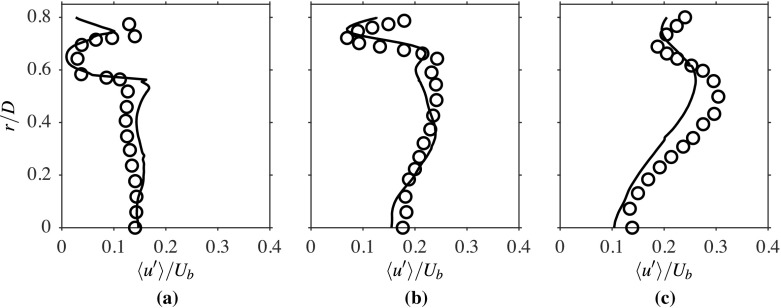
Computed (−−−) and measured [1] (∘) radial variations of $\langle { u }^{\prime } \rangle / { U }_{b }$ at **a** *x*/*D* = 0.2, **b** 0.8 and **c** 1.6 for the reacting case

**Fig. 11:**
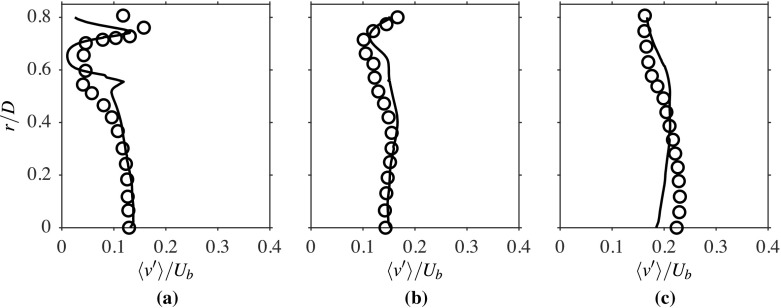
Computed (−−−) and measured [1] (∘) radial variations of $\langle { v }^{\prime } \rangle / { U }_{b }$ at **a** *x*/*D* = 0.2, **b** 0.8 and **c** 1.6 for the reacting case

The rms of turbulent fluctuations, *u*^′^ and *v*^′^, are shown in Figs. 10 and 11 respectively. Since the turbulence is shear driven in this burner, it is expected to see peak values of *u*^′^/*U*_*b*_ and *v*^′^/*U*_*b*_ in the regions of strong shear, which is observed in the computations, as seen in these two figures. However, the inner peak for *u*^′^/*U*_*b*_ is somewhat lower than the outer peak for the location *x*/*D* = 0.2. This could be due to the additional shear generated by the entraining flow in the outer shear layer. Also, the reason for the drop in *v*^′^/*U*_*b*_ for the region 0.4 ≤ *r*/*D* ≤ 0.6 at the location *x*/*D* = 0.2 is not clear. It is indeed expected to see a peak, as suggested by the computational result, for the variation of *u*^′^/*U*_*b*_ in the region close to the bluff body base and shows a similar trend to the isothermal case. The radial variations of both quantities, *u*^′^/*U*_*b*_ and *v*^′^/*U*_*b*_, are well captured, except for some over predictions within the inner shear layer. The variations are well captured at the second location, as shown in Figs. 10b and 11b, but the computation under predicts *u*^′^/*U*_*b*_ and *v*^′^/*U*_*b*_ within the recirculation zone at *x*/*D* = 1.6, as shown in Figs. 10c and 11c. Nonetheless, the overall agreement of these quantities with the experimental data is good.

### Discussion

**Fig. 12:**
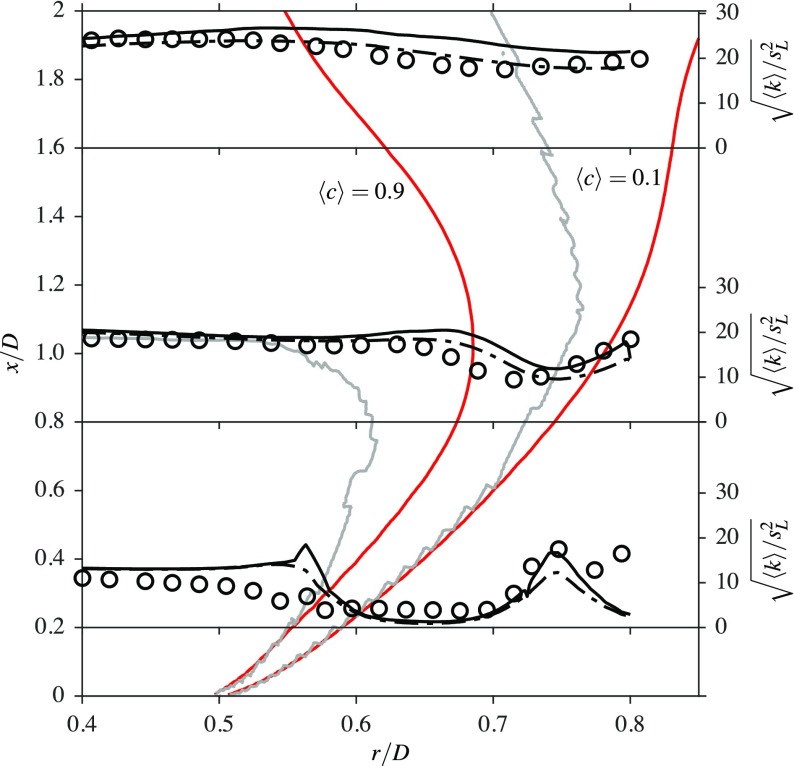
Radial variations of the computed and measured turbulent kinetic energy in the reacting case. The edges of the inner shear layer are shown with () and those of the flame brush, marked using $\left \langle \widetilde {c}\right \rangle = 0.1$ and 0.9, are shown with (). The turbulent kinetic energy with (−−−) and without (− ⋅ −) the modelled SGS contribution are also shown

It has been shown that the flame and flow features are well captured by the LES, showing good comparisons with the measurements to a similar standard reported in previous numerical studies using different combustion models [18–20]. In general, the magnitudes of *u*^′^ and *v*^′^ increase with axial distance from the bluff body base, suggesting that the turbulent kinetic energy production increases with axial distance. The turbulent kinetic energy at the axial locations *x*/*D* = 0.2, 0.8 and 1.6 analysed so far is computed using both the experimental data and LES. Since *w*^′^ is not available from the measurements [1], the turbulent kinetic energy is estimated as 〈*k*〉_exp_ = 0.5(*u*^′^^2^ + 2*v*^′^^2^) by assuming that *w*^′^≃ *v*^′^. This is compared to the turbulent kinetic energy from the LES results, computed as 〈*k*〉_res_ = 0.5(*u*^′^^2^ + *v*^′^^2^ + *w*^′^^2^), using only the resolved velocities, and also the total kinetic energy 〈*k*〉_tot_ = 〈*k*〉_res_ + 〈*k*〉_sgs_ by including the SGS kinetic energy ${ k }_{\mathrm { sgs } } = 3 { { u }_{{\Delta } }^{\prime } }^{2 } / 2$. These variations are shown in Fig. 12, where the symbols are for 〈*k*〉_exp_, the black line represents 〈*k*〉_tot_ and the dash-dotted black line is for 〈*k*〉_res_. It is worth noting that the PIV measurements may lack the velocity fluctuations that are smaller than the size of the interrogation region, if the interrogation window is larger than several times the Kolmogorov length.^1^ Grey lines represent the shear layer edges originating from the bluff body and red lines denote the flame brush, which is marked using $\langle \widetilde { c }\rangle = 0.1$ and 0.9. The edges of the shear layer are drawn using the contours at 10% of the local peaks of $\partial \langle \widetilde { U } \rangle / \partial r$.

It is seen that the sharp peak of the total kinetic energy at *x*/*D* = 0.2 is located within the inner shear layer, where the flame brush is also located. It should be noted that the other sharp peak is within the outer shear layer and is not considered for the analysis here, as it is outside the flame brush. Since this peak is not present for 〈*k*〉_res_, this means that there are some flame generated velocity fluctuations in this region. These fluctuations can come from two sources; one is flame generated turbulence and the other one is due to intermittent effects from the flame. The current framework for the analysis makes it challenging to ascertain these two mechanisms and to identify a dominant one; this will be explored in a future study. Since the numerical grid used here resolves more than 80*%* of the turbulence, the SGS turbulence is expected to be small and flame induced effects would be strong, as noted above. Nevertheless, the trends are the same for both 〈*k*〉_res_ and 〈*k*〉_tot_. Furthermore, it should be noted that *w*^′^≃ *v*^′^ is assumed for the experimental data and the validity of this approximation is an open question. At *x*/*D* = 0.8 and 1.6, it is observed that the peak value of 〈*k*〉_res_ is within the shear layer and decays from the product side to the reactant side, for the burner configuration used here. There is a slight increase close to the reactant side for these two locations, which is caused by the outer shear layer resulting from ambient air entrainment. Also, it is observed that there is some interaction between the flame and inner shear layer. Although the entire shear layer is within the flame brush near the flame stabilisation region, this layer moves out of the flame gradually when moving downstream. The contour of $\langle \widetilde { c } \rangle = 0.9$ moves from the inner to outer side of the shear layer starting from the bluff body base to *x*/*D* = 1, as shown in Fig. 12. This contour then moves back into the shear layer in a similar manner to the outer part of the shear layer. On the other hand, the $\langle \widetilde { c } \rangle = 0.1$ contour continuously moves further away from the inner shear layer from *x*/*D* = 0.6 because the reactant stream is unconfined. This relative movement of the shear layer and the flame brush will lead to an unexpected behaviour of turbulent kinetic energy across the flame brush for various downstream positions.

**Fig. 13:**
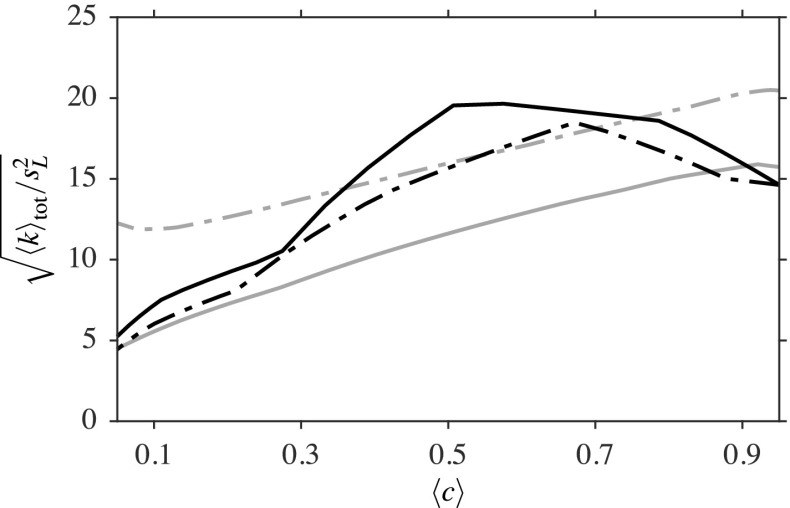
Kinetic energy variation across the flame brush at four streamwise locations; *x*/*D* = 0.1 (−−−), 0.2 (− ⋅ −), 0.4 () and 0.8 ()

A previous DNS study [44] reported that the turbulent kinetic energy peaks within the flame for the corrugated flamelet regime, but increases across the flame from the reactant to product side of the flame for the thin reaction zones regime. These behaviours are seen in the variations of 〈*k*〉_tot_ with $\langle \widetilde { c } \rangle $ and are depicted in Fig. 13. It is seen that the peak turbulent kinetic energy is at the centre of the flame for *x*/*D* = 0.1 and then shifts towards the product side for *x*/*D* = 0.2 and *x*/*D* = 0.4. However, there is no peak for the location *x*/*D* = 0.8, which suggests that there is a transition in the combustion regimes in the region 0.2 < *x*/*D* < 0.8. The flame is burning within the corrugated flamelet regime close to the bluff body, which is shown by the flame wrinkling caused by large scale eddies resulting from the Kelvin-Helmholtz instability, and this thin layer structure is seen in Fig. 5. Hence, it could be said that the flame near the stabilisation region is quasi-steady and quasi-laminar. This claim is also supported by the high values of the parameter $\widehat { \mathrm { Da } }_{{\Delta } }$ (indicating stronger reaction rates) in those regions, as observed in Fig. 5.

**Fig. 14:**
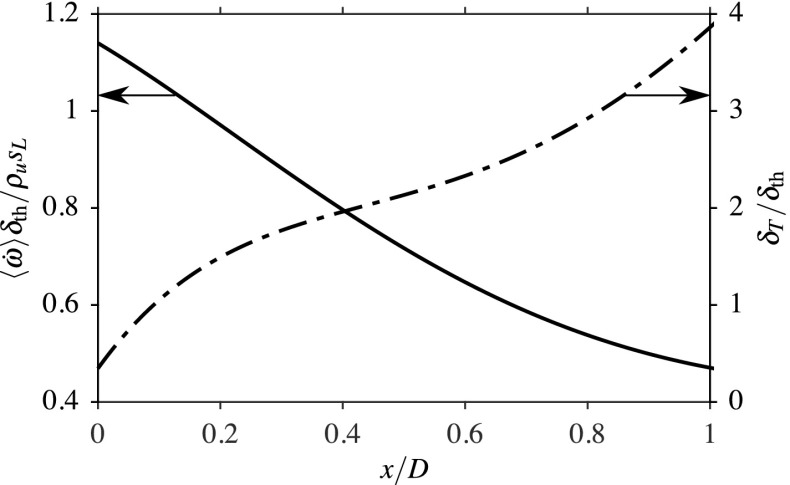
Variations of the peak time-averaged reaction rate (−−−) and flame thickness (− ⋅ −) with axial distance

To further investigate this quasi-laminar flame observation, the variation of the peak averaged reaction rate, $\langle \overline { \dot { \omega } } \rangle $, and the flame brush thickness, ${ \delta }_{T } = 1 / { \vert \partial \langle \widetilde { c } \rangle / \partial r \vert }_{\mathrm { max } }$, are studied. If the flame is truly laminar, then the peak reaction rate should scale as *ρ*_*u*_*s*_*L*_/*δ*_th_, as laminar flame theory suggests. Therefore, the peak averaged reaction rate, normalised using this scaling, should be of order unity if the flame is quasi-laminar, and *δ*_*T*_/*δ*_th_ must also be of order unity. These two normalised quantities are plotted in Fig. 14 as a function of *x*/*D*. The strain thinning of the filtered flame, caused by large scale eddies near the flame base, yields the normalised reaction rate to be larger than unity. Furthermore, it is shown that *δ*_*T*_/*δ*_th_ < 1 for *x*/*D* ≤ 0.2, which supports the quasi-laminar flame observation.

**Fig. 15:**
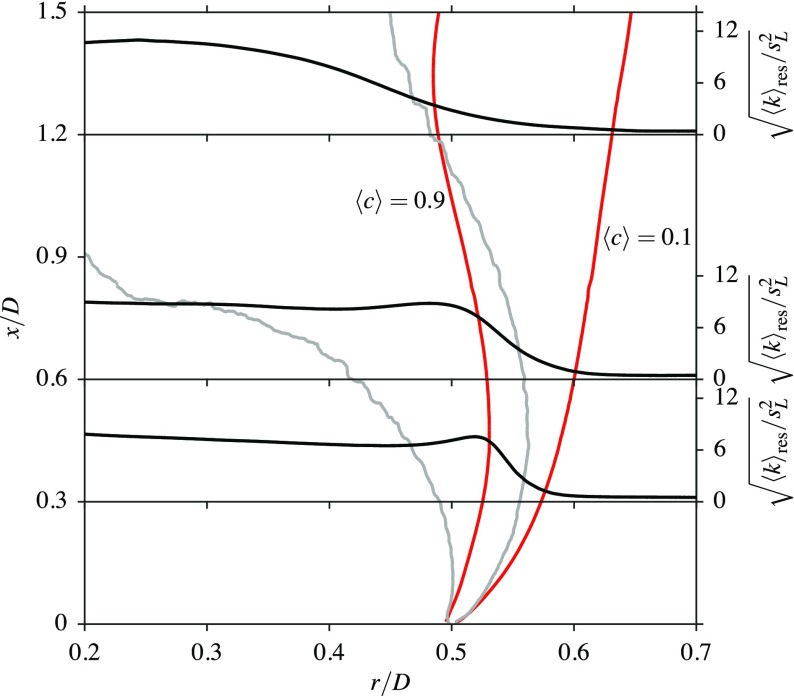
Radial variations of the turbulent kinetic energy in a confined bluff body stabilised flame with 2*%* incoming turbulence. The edges of the inner shear layer are shown with () and those of the flame brush, marked using $\langle \widetilde {c}\rangle = 0.1$ and 0.9, are shown with ().

**Fig. 16:**
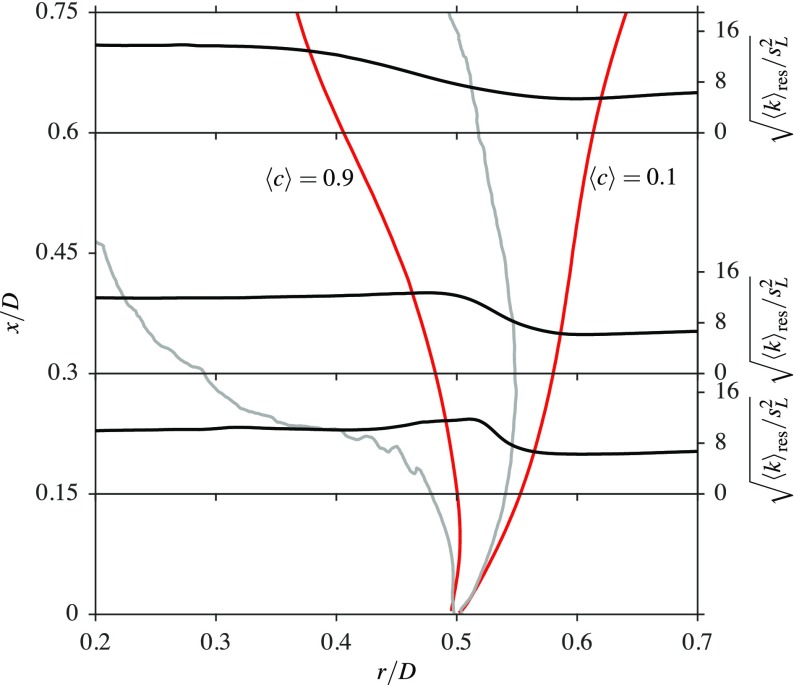
Radial variations of the turbulent kinetic energy in a confined bluff body stabilised flame with 22% incoming turbulence. The edges of the inner shear layer are shown with () and those of the flame brush, marked using $\langle \widetilde {c}\rangle = 0.1$ and 0.9, are shown with ()

Figures 15 and 16 show the radial variation of 〈*k*〉_res_, normalised by ${ s }_{L }^{2}$, for three axial locations inside the recirculation zones of confined bluff body stabilised flames; this configuration has been investigated in an earlier study [11]. These two flames are denoted as CF02 and CF22 for the following discussion. For this study, both of these flames have an equivalence ratio of *ϕ* = 0.8, which is close to the value used for flame A1, as discussed previously. Flame CF02 has a turbulence intensity of approximately *u*^′^/*U*_*b*_ = 2*%* at the base of the bluff body, whereas flame CF22 has a turbulence intensity of approximately *u*^′^/*U*_*b*_ = 22*%*. The lengths of the recirculation zones are 1.37*D* and 0.73*D* for flames CF02 and CF22 respectively, where it is seen that these recirculation zone lengths are significantly shorter than the length of 2.02*D* observed for flame A1. As noted in Section 1, the aim of the following discussion is to distinguish the behaviour between flame A1 (open flame) and flames CF02 and CF22 (confined flames). Flame A1 has only shear driven turbulence, with no incoming turbulence, but the other two flames have both shear driven and incoming turbulence.

It is clear that the relative locations of the shear layers and flame brush are very similar near the bluff body for these three flames, as seen when comparing Figs. 12, 15 and 16. In the low turbulence level case in Fig. 15, the initial evolution of the shear layer is similar to that observed for the flame A1 in Fig. 12. However, the $\langle \widetilde { c } \rangle = 0.9$ contour moves completely out of the shear layer at around *x*/*D* = 1.18 which is approximately 86*%* of the recirculation zone length. It can be suggested that beyond this point, the flame does not experience any shear generated turbulence. This is not the case with flame A1, as the absence of walls allows the flame to remain within the shear layer, whereas the shear layer is pushed further inwards for the confined flames. The turbulent kinetic energy increases across the flame, since the combustion is in the corrugated flamelets regime, as noted by Langella et al. [11]. This behaviour of the turbulent kinetic energy is consistent with many past DNS studies using statistically planar premixed flames.

When the incoming turbulence intensity is increased to 22*%*, it is shown that part of the shear layer always remains within the flame for the entire recirculation zone length, as illustrated in Fig. 16, which is also the case for flame A1. However, flame CF22 experiences more shear generated turbulence than CF02, as the flame brush and shear layer are thicker, and hence there is more of an overlap for flame CF22. The increasing trend of the turbulent kinetic energy across the contour 〈*c*〉 = 0.1 for flame A1 is due to the outer shear layer resulting from the air entrainment. The flame stabilisation mechanisms (the relative positions of the shear layer and the flame brush in the vicinity off the bluff body base) are similar for both confined flames as the shear layer is pushed inwards very close to the bluff body from approximately *x*/*D* = 0.1 and *x*/*D* = 0.05 for flames CF02 and CF22 respectively. This is not the case for flame A1, as both the product and reactant sides of the flame are well aligned with the shear layer until *x*/*D* = 0.2. The reactant side of the flame remains aligned with the outer part of the shear layer from this point until *x*/*D* = 0.6, as seen in Fig. 12. Thus, the presence of the walls close to the bluff body does affect the relative positions of the flame and shear layer. Although a strained flamelet model may capture this stabilisation with some improved accuracy, the framework of the unstrained flamelet model used here has previously given a better overall performance compared to a strained flamelet model [24] and for a bluff body flame [11].

## Summary and Conclusion

A lean turbulent premixed bluff body stabilised flame far from blow-off conditions is computed and compared against the experimental data. The filtered reaction rate is modelled using an unstrained premixed flamelet closure with a presumed sub-grid PDF for the reaction progress variable. Both the filtered progress variable and its corresponding SGS variance are obtained using their transport equations. An algebraic expression involving both turbulent and chemical time scales is used to model the SGS scalar dissipation rate. The computational model is validated first using its isothermal case. The recirculation zone behind the bluff body is well captured in terms of its length, as well as the variations of flow field statistics, which are evaluated by direct comparisons with PIV measurements. The location of the inner shear layer and the distribution of the axial rms velocity in that region are also well captured.

These statistics from the LES are compared with measurements for a stable flame and it is shown that the recirculation zone and inner shear layers are accurately captured in the LES. In addition, the overall shape of the flame in the LES is slightly longer and wider than that observed in the experiment. The highest SGS Damköhler number is located close to the bluff body base, which is caused by the largest filtered reaction rate. Further analysis of the distribution of the turbulent kinetic energy across the flame and inner shear layer showed that the turbulent kinetic energy has a peak value within the flame in the region close to the bluff body base. However, the turbulent kinetic energy increases from the reactant to product side of the flame further downstream. This suggests a shift from the corrugated flamelets regime to the thin reaction zones regime of turbulent premixed combustion. This observation also suggests that the flame exhibits quasi-laminar behaviour close to the bluff body base, which is corroborated further by analysing the computed filtered reaction rate and flame brush width. The behaviour of the open bluff body stabilised flame is compared to confined bluff body stabilised flames, which experienced additional incoming turbulence. The main difference between these flames is observed to be the relative positioning of the shear layer and flame brush and their spatial evolution.

## Footnotes

1 Suggested by an anonymous reviewer
